# Structural basis for pore blockade of human voltage-gated calcium channel Ca_v_1.3 by motion sickness drug cinnarizine

**DOI:** 10.1038/s41422-022-00663-5

**Published:** 2022-04-27

**Authors:** Xia Yao, Shuai Gao, Nieng Yan

**Affiliations:** Department of Molecular Biology, Princeton University, Princeton, NJ USA

**Keywords:** Cryoelectron microscopy, Calcium signalling

Dear Editor,

Voltage-gated calcium (Ca_v_) channels regulate a broad range of physiological processes, such as muscle contraction, synaptic signal transduction, and hormone secretion.^1^ Ca_v_1.3 channels belong to the L-type (L for long-lasting current) Ca_v_ subfamily (LTCC) and display diverse tissue distributions in brain, ear, heart, and endocrine organs.^2^ Of note, Ca_v_1.3 channels are predominantly expressed in cochlea inner hair cells, whose normal development and synaptic transmission are crucial for proper auditory function.^3^

Therapeutic agents targeting Ca_v_1.3 channels hold promises for Parkinson’s disease, resistant hypertension, and hearing dysfunction.^4^ In addition to the widely prescribed dihydropyridine (DHP), phenylalkylamine (PAA), and benzothiazepine (BTZ) drugs, Ca_v_1.3 channels are subject to regulation by compounds with the diphenylmethylpiperazine (DPP) core group, exemplified by cinnarizine and flunarizine (Supplementary information, Fig. S1).^5^ DPP analogs, with their dual activities as antihistamines and Ca_v_ channel blockers, are used to treat motion sickness, although the molecular details for their mode of actions (MOA) remain to be revealed.^6^

The rabbit Ca_v_1.1 multimeric complex purified from endogenous skeletal muscle tissues has been employed as a prototype for structural analysis of LTCC antihypertensive and antiarrhythmic agents.^7^ Here, we present the high-resolution structures of human Ca_v_1.3 complex, comprising the pore-forming α1 and auxiliary α2δ-1 and β3 subunits. Ligand-free and cinnarizine-bound human Ca_v_1.3 structures were determined at 3.0 Å and 3.1 Å resolutions, respectively.

To attain heterotrimeric Ca_v_1.3 proteins, we transfected HEK293F cells with plasmids encoding full-length α1, α2δ-1 and β3 subunits at the mass ratio of 1.5:1.2:1. After tandem purification through affinity and size exclusion chromatography, peak fractions containing all three subunits were pooled for cryo-sample preparation (Supplementary information, Fig. S2a). The Ca_v_1.3–drug samples were prepared by incubating concentrated proteins in the presence of 250 μM cinnarizine or 150 μM cp-PYT (compound 8 in^8^), a pyrimidine-2,4,6-triones derivative that showed modest selective inhibition against Ca_v_1.3.

While cinnarizine is unambiguously resolved (Fig. 1a), there is no discernible density for cp-PYT in the electron microscopy (EM) map. cp-PYT was predicted to occupy the DHP-binding site,^7,9,10^ which is on the interface of repeats III and IV in the pore domain (PD), known as the III–IV fenestration. As the entire PD is clearly resolved, the invisibility of cp-PYT is unlikely to result from potential flexibility. cp-PYT was shown to be a modest inhibitor of Ca_v_1.3, with the IC_50_ > 50 μM.^11,12^ It is possible that addition of 150 μM cp-PYT did not warrant a stable drug–channel complex that survived cryo-sample preparation. We will refer  to the structure of Ca_v_1.3 treated with cp-PYT as the apo state (Supplementary information, Figs. S2–S4 and Table S1).

**Fig. 1:**
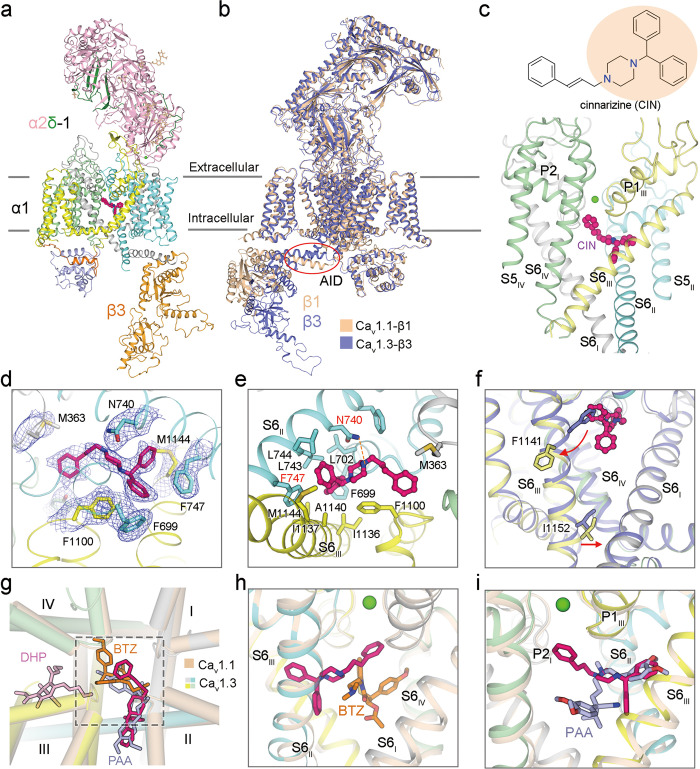
Pore blockade of human Ca_v_1.3 by cinnarizine. **a** Overall structure of Ca_v_1.3 bound to cinnarizine. The complex comprises transmembrane α1 (gray, cyan, yellow, and pale green for Repeats I–IV, respectively), extracellular α2δ-1 (light pink for α2, and green for δ), and cytosolic β3 subunits (bright orange). Cinnarizine and Ca^2+^ ions are shown as magenta, and green spheres. Wheat sticks indicate *N*-glycans on the α2δ-1 subunit. **b** Superimposition of rabbit Ca_v_1.1 complex (colored wheat, PDB: 5GJV) and human Ca_v_1.3 complex (colored purple). Despite treatment with 150 μM cp-PYT before cryo-sample preparation, there is no density for the drug in the EM map of Ca_v_1.3. This structure will thus serve as a reference to study the conformational changes upon cinnarizine binding, as shown in panel **f**. Red circle highlights the minor structural shift at the AID. **c** Cinnarizine is accommodated in the central cavity of the PD. Chemical structure of cinnarizine is shown, with the core DPP group shaded light orange. **d** EM map for cinnarizine and surrounding residues. The densities, shown as blue meshes, are contoured at 4 σ in PyMol. **e** Molecular details for cinnarizine coordination. A potential hydrogen bond is indicated by orange dashes. **f** Local conformational changes upon cinnarizine binding. Red arrows indicate the structural shift of S6_III_ upon cinnarizine binding. **g** The binding mode of cinnarizine is different from that of DHP drugs and other pore blockers. Shown here is an extracellular view of superimposed structures of Ca_v_1.3 (domain-colored) bound to cinnarizine and Ca_v_1.1 (colored wheat) bound to amlodipine (DHP drug, pink sticks, PDB: 7JPX), diltiazem (BTZ, orange sticks, PDB: 6JPB), and verapamil (PAA, light blue sticks, PDB: 6JPA). **h** Orthogonal binding pockets for cinnarizine and BTZ. **i** PAA partially overlaps with cinnarizine.

Similar to rabbit Ca_v_1.1, the conformation of human Ca_v_1.3 is consistent with an inactivated state, as all voltage-sensing domains (VSDs) are depolarized and the intracellular gate of the PD is closed (Supplementary information, Fig. S5). The structures of the α1 subunit in Ca_v_1.3 and Ca_v_1.1 can be superimposed with the root-mean-square deviation (RMSD) of 1.63 Å over 1017 Cα atoms (Fig. 1b; Supplementary information, Fig. S6). A minor structural difference occurs in the α1-interacting domain (AID), which is responsible for the association of β subunit to α1 and serves as a lever for β subunit to regulate the gating properties of Ca_v_ channels.^13,14^

Cinnarizine is accommodated in the central cavity of PD. The structure thus reveals that the motion sickness drugs are pore blockers of Ca_v_ channels (Fig. 1c, d). The entire molecule nestles right beneath the selectivity filter (SF), directly obstructing the ion-conducting pathway. Cinnarizine is coordinated through extensive contacts between its core DPP group and residues from repeats II and III. The diphenylmethane group occupies a hydrophobic pocket composed of Phe699, Leu702, Leu743, Leu744 and Phe747 in repeat II, and Ile1136, Ile1137, Ala1140 and Met1144 in repeat III. Among these residues, Phe747 on S6_II_ appears to make the primary contribution via π–π stacking with the two phenyl rings of cinnarizine. Besides, piperazine of the core group is H-bonded to Asn740 on S6_II_. The cinnamyl group, which points to the SF vestibule, is sandwiched by Met363 and Phe1100, whose carbonyl oxygen groups are constituents of the inner Ca^2+^-binding site in the SF (Fig. 1e).

Compared to the apo state, the S6 segments in the four repeats undergo structural shifts upon cinnarizine binding (Fig. 1f; Supplementary information, Fig. S7). To accommodate cinnarizine, the helical turn of S6_III_ containing Phe1141 undergoes both a π → α secondary structural transition and an outward motion to avoid steric clash. Interestingly, the helical turn where the gating residue Ile1152 is positioned undergoes an α → π transition, which appears to tighten the intracellular gate (Supplementary information, Fig. S8).

The binding mode of cinnarizine is distinct from those of DHP allosteric drugs and previously reported pore blockers.^4^ When structures of Ca_v_1.1 in the presence of amlodipine (DHP drug), diltiazem (BTZ), and verapamil (PAA) are separately aligned with that of cinnarizine-bound Ca_v_1.3, two major binding pockets are revealed to accommodate the diverse LTCC modulators (Fig. 1g). DHP drugs, consistent with their nature of being allosteric antagonists and agonists, occupy the III–IV fenestration, a pocket separated from that for pore blockers. Diltiazem, verapamil, and cinnarizine all directly occlude the ion-permeation pathway, but with different coordination characteristics.

The binding site for cinnarizine is orthogonal to that for diltiazem. The core benzothiazepine group of diltiazem is at a lower position compared to the cinnamyl group of cinnarizine. The methoxyphenyl group of diltiazem is supported by residues from repeats IV (Fig. 1h). In comparison, cinnarizine and verapamil partially overlap in the central cavity, interacting mainly with the hydrophobic residues on S6_II_ and S6_III_ (Fig. 1i). The cinnamyl group of cinnarizine directly inserts into the SF vestibule, whereas the moiety of verapamil responsible for pore blockage is positioned at the center of the pore cavity.

Taken together, DPP, BTZ and PAA compounds are all pore blockers of LTCC. To stabilize the coordination in channel pores, one end of the small molecules requires extensive hydrophobic interactions with S6 helices, S6_IV_ for BTZ, and both S6_II_ and S6_III_ for PAA and DPP. The other end can be positioned at variable positions in the central cavity.

In sum, the structure of human Ca_v_1.3 channel bound to cinnarizine unveils a direct pore blockade of LTCC by motion sickness drugs. Local structural shifts at the cinnarizine-binding site cause an axial rotation of the ensuing helical segment. The α → π transition of the gating residue-locating helical turn in S6_III_ further constricts the ion-permeation pore. The structures shown here, along with our previous studies, reveal the molecular details for the MOA of diverse Ca_v_ channel modulators and lay the foundation for structure-aided drug discovery. Furthermore, all the reported structures of LTCC, either Ca_v_1.1 from endogenous tissue or Ca_v_1.3 from heterologous expression, are featured with four up VSDs and closed PD, consistent with inactivated states. In contrast, the recently reported structures of N-type Ca_v_2.2 channels reveal a down VSD_II_.^14,15^ Establishment of a recombinant expression system for L-type Ca_v_ channels paves the avenue to investigate the intrinsic biophysical differences between different types of Ca_v_ channels.

## Supplementary information

supplementary information, Figures and Table

## Data Availability

The atomic coordinates and EM maps for cinnarizine-bound and apo Ca_v_1.3 have been deposited in the Protein Data Bank with the accession codes 7UHF (cinnarizine-bound Ca_v_1.3) and 7UHG (apo Ca_v_1.3) and in the Electron Microscopy Data Bank with the codes EMD-26513 (cinnarizine-bound Ca_v_1.3) and EMD-26514 (apo Ca_v_1.3), respectively.
