# Supplementary material for: Structural basis for pore blockade of human voltage-gated calcium channel Cav1.3 by motion sickness drug cinnarizine
Source: Cell Res. 2022 Apr 27;32(10):946–8. doi: 10.1038/s41422-022-00663-5 (PMC9525318; doi:10.1038/s41422-022-00663-5)
Supplement: Supplementary file 1 — supplementary information, Figures and Table [file 41422_2022_663_MOESM1_ESM.pdf]

Supplementary Information

Structural basis for pore blockade of human voltage-gated calcium channel $Ca_v1.3$ by motion sickness drug cinnarizine

Xia Yao[1,2,*], Shuai Gao[1,2], Nieng Yan[1,*]

[1]Department of Molecular Biology, Princeton University, Princeton, NJ 08544, USA

[2]These authors contributed equally: Xia Yao, Shuai Gao.

*To whom correspondence should be addressed: Nieng Yan (nyan@princeton.edu);

Xia Yao (xiayao@princeton.edu)

Materials and Methods

Transient expression of human $Ca_v1.3$ complex in HEK293F cells

Codon-optimized cDNAs of *CACNA1D* for full-length $Ca_v1.3$ α1 (2,161 residues, Uniprot Q01668-1), *CACNA2D1* for α2δ-1 (1,103 residues, Uniprot P54289-1), and *CACNB3* for β3 (484 residues, Uniprot P54284-1) were synthesized (BGI Geneland Scientific) and subcloned into pCAG vector with tandem twin-strep and Flag tags at the amino (N) terminus for α1 subunit, N-terminal Flag tag and C-terminal His tag for β3 subunit. HEK293F suspension cells (Thermo Fisher Scientific, R79007) were cultured in Freestyle 293 medium (Thermo Fisher Scientific) at 37 °C, supplied with 5% CO_2 under 60% humidity. When cell density reached 1.5–2.0 × 10^6 cells per mL, plasmid mixture including 0.75 mg α1, 0.6 mg α2δ-1 and 0.5 mg β3, and 3 mg polyethylenimine (Polysciences) were added into the cell culture for transient expression of human $Ca_v1.3$ complex.

Protein purification of human $Ca_v1.3$ and complex preparation with cinnarizine

For one batch of protein purification, 8 liters of HEK293F cells were collected approximately 72 h after transfection by centrifugation at 3,600 g for 10 min. The pellets were resuspended in the lysis buffer containing 25 mM HEPES (pH 7.4), 150 mM NaCl, 2 mM $CaCl_2$ and the protease inhibitor cocktail containing 2.6 μg mL^{-1} aprotinin (VWR Life Science) and 1.4 μg mL^{-1} pepstatin (VWR Life Science). The suspension was supplemented with glyco-diosgenin (GDN, Anatrace) to a final concentration of 1% (w/v), n-dodecyl-β-D-maltopyranoside (DDM, Anatrace) to 0.2% (w/v), and cholesteryl hemisuccinate Tris salt (CHS, Anatrace) to 0.04% (w/v). After incubation at 4 °C overnight, the mixture was centrifuged at 35,000 g for 30 min, and the supernatant was applied to anti-Flag M2 affinity resin (Sigma). The resin was rinsed

with wash buffer (buffer W) containing 25 mM HEPES (pH 7.4), 150 mM NaCl, 2 mM $CaCl_2$ and 0.01% GDN. After elution with buffer W plus 0.2 mg mL^{-1} Flag peptide (synthesized by GenScript), the eluent was concentrated using a 100-kDa cut-off Amicon (Millipore) and further purified through size-exclusion chromatography (Superose 6 10/300 GL, GE Healthcare) that was pre-equilibrated in buffer W. The peak fractions were pooled and concentrated to 15-20 mg mL^{-1} for cryo-sample preparation.

For structural determination of Ca_v1.3-drug complexes, cp-PYT (Aobious) and cinnarizine (MedChemExpress) were separately added to the concentrated protein solution at a final concentration of 150 μM and 250 μM, respectively, and incubated at 4 °C for 30 min before making cryo-grids.

Cryo-EM sample preparation and data collection

Aliquots of 3.5 μL protein-drug mixtures were loaded onto glow-discharged holey carbon grids (Quantifoil Au R1.2/1.3 or Cu R1.2/1.3), which were blotted for 6 s and plunge-frozen in liquid ethane cooled by liquid nitrogen using a Vitrobot Mark IV (Thermo Fisher) at 8 °C with 100% humidity. Grids were transferred to a Titan Krios electron microscope (Thermo Fisher) operating at 300 kV and equipped with a Gatan Gif Quantum energy filter (slit width 20 eV) and spherical aberration (Cs) image corrector. Micrographs were recorded using a K2 Summit counting camera (Gatan) in super-resolution mode with a nominal magnification of 105,000×, resulting in a calibrated pixel size of 0.557 Å. Each stack of 32 frames was exposed for 5.6 s, with an exposure time of 0.175 s per frame. The total dose for each stack was about 50 e^- per $Å^2$. SerialEM was used for fully automated data collection[1]. All 32 frames in each stack were

aligned, summed and dose-weighted using MotionCorr2[2] and two fold-binned to a pixel size of 1.114 Å per pixel. The defocus values were set from −1.9 to −2.1 μm and estimated by Gctf.[3]

Image processing

For simplicity, we will refer to samples with cp-PYT and cinnarizine as Ca_v1.3-PYT and Ca_v1.3-CIN, respectively. However, no cp-PYT was observed in the structure.

A total of 2,144 (Ca_v1.3-PYT) and 3,898 (two batches of data were collected for Ca_v1.3-CIN, 1st: 1,870; 2nd: 2,028) cryo-EM micrographs were collected. 1,383,355 (Ca_v1.3-PYT) and 2,621,186 (Ca_v1.3-CIN, 1st: 1,379,686; 2nd: 1,241,500) particles were auto-picked by RELION-3.0[4] using 2D classes of rabbit Ca_v1.1 (EMD-22426)[5] in the side and tilted views as references. All subsequent 2D and 3D classification and refinement were performed with RELION-3.0. One round of reference-free 2D classification was performed to remove ice spots, contaminants, and aggregates, yielding 1,332,220 and 2,397,544 (1st: 1,234,028; 2nd: 1,163,516) particles for Ca_v1.3-PYT and Ca_v1.3-CIN, respectively.

For Ca_v1.3-PYT, bin2 particles were processed by a global search with K = 1 to determine the initial orientation alignment parameters. The EM map of human Ca_v2.2 (EMD-23868)[6] that low-pass filtered to 20 Å was used as an initial reference. Output for the 35th-40th iterations of global classification was further applied to the local angular search 3D classification with four classes. A total of 459,833 particles were selected by combining the good classes of the local angular search 3D classification. The particles were then re-extracted with box size of 280 and pixel size of 1.114 Å. These particles yielded a reconstruction at 3.4 Å after 3D auto-refinement with an adapted mask. At last, skip align 3D classification using bin1 particles after Bayesian polishing afforded final reconstruction at 3.0 Å from 91,481 particles.

For $Ca_v1.3$-CIN, 1,234,028 particles (selected from 2D classes) of the first dataset were further processed following the same procedure as for $Ca_v1.3$-PYT. The output of the 35th–40th iterations was further applied to local angular search 3D classification with four classes. A total of 510,226 particles were selected by combining the good classes of local 3D classification. The particles were then re-extracted using a box size of 280 and pixel size of 1.114 Å. These particles yielded a reconstruction at 4.2 Å after 3D auto-refinement with an adapted mask. Skip align 3D classification using bin1 particles after Bayesian polishing resulted in a reconstruction at 3.4 Å from 60,440 particles. For the second dataset, 1,163,516 particles were re-extracted to bin2 particles and processed with a global search with K = 4 using multi-reference (4 references) 3D classification. The good class of the 10th iteration was selected and further applied to local angular search 3D multi-reference classification with K = 4. A total of 246,144 particles were selected by combining the good classes of the local angular search 3D multi-reference classification. The particles were then re-extracted using a box size of 280 and pixel size of 1.114 Å. These particles yielded reconstructions at 3.9 Å after 3D auto-refinement with an adapted mask. Skip align 3D classification using bin1 particles after Bayesian polishing resulted in a reconstruction at 3.3 Å from 46,852 particles. At last, combined particles from two datasets were applied to skip align 3D classification, affording the final reconstruction at 3.1 Å out of 51,625 particles.

All 2D classification, 3D classification and 3D auto-refinement were performed with RELION 3.0. Resolutions were estimated using the gold-standard Fourier shell correlation 0.143 criterion with high-resolution noise substitution[7,8].

Model building and refinement

Model building of $Ca_v1.3$ was based on the reported $Ca_v1.1$ and $Ca_v2.2$ structures. The initial model of $Ca_v1.3$ α1 subunit was built in SWISS-MODEL[9] based on the structure of rabbit $Ca_v1.1$ (PDB code 5GJV)[10], and that of α2δ-1 and β3 were based on the structure of $Ca_v2.2$ (PDB code 7MIY)[6]. We docked α1-interacting guanylate kinase domain and distal domain of β3 subunit into $Ca_v1.3$ EM map as a rigid body. The initial model was then manually docked into the 3.0 Å $Ca_v1.3$-PYT 3D map in Chimera[11] and manually adjusted in COOT[12], followed by refinement against the corresponding maps by phenix.real_space_refine program in PHENIX[13] with secondary structure and geometry restraints. For model building of the $Ca_v1.3$-CIN, the coordinates for α1, α2δ-1 and β3 from apo-$Ca_v1.3$ were docked into the 3.1 Å $Ca_v1.3$-CIN map separately, followed by manual adjustment in COOT.

Statistics of the map reconstruction and model refinement can be found in Table S1. All structure figures were prepared in ChimeraX[14] and PyMol[15].

References

1 Mastronarde, D. N. Automated electron microscope tomography using robust prediction of specimen movements. *J. Struct. Biol.* **152**, 36-51, doi:10.1016/j.jsb.2005.07.007 (2005).

2 Zheng, S. Q. *et al.* MotionCor2: anisotropic correction of beam-induced motion for improved cryo-electron microscopy. *Nat Methods* **14**, 331-332, doi:10.1038/nmeth.4193 (2017).

3 Zhang, K. Gctf: Real-time CTF determination and correction. *J. Struct. Biol.* **193**, 1-12, doi:10.1016/j.jsb.2015.11.003 (2016).

4 Zivanov, J. *et al.* New tools for automated high-resolution cryo-EM structure determination in RELION-3. *Elife* **7**, e42166, doi:10.7554/eLife.42166 (2018).

5 Gao, S. & Yan, N. Structural Basis of the Modulation of the Voltage-Gated Calcium Ion Channel Ca(v) 1.1 by Dihydropyridine Compounds*. *Angew. Chem. Int. Ed. Engl.* **60**, 3131-3137, doi:10.1002/anie.202011793 (2021).

6 Gao, S., Yao, X. & Yan, N. Structure of human Ca(v)2.2 channel blocked by the painkiller ziconotide. *Nature* **596**, 143-147, doi:10.1038/s41586-021-03699-6 (2021).

7 Rosenthal, P. B. & Henderson, R. Optimal determination of particle orientation, absolute hand, and contrast loss in single-particle electron cryomicroscopy. *J. Mol. Biol.* **333**, 721-745, doi:10.1016/j.jmb.2003.07.013 (2003).

8 Chen, S. *et al.* High-resolution noise substitution to measure overfitting and validate resolution in 3D structure determination by single particle electron cryomicroscopy. *Ultramicroscopy* **135**, 24-35, doi:10.1016/j.ultramic.2013.06.004 (2013).

9 Waterhouse, A. *et al.* SWISS-MODEL: homology modelling of protein structures and complexes. *Nucleic Acids Res.* **46**, w296-w303, doi:10.1093/nar/gky427 (2018).

10 Wu, J. *et al.* Structure of the voltage-gated calcium channel Ca(v)1.1 at 3.6 Å resolution. *Nature* **537**, 191-196, doi:10.1038/nature19321 (2016).

11 Pettersen, E. F. *et al.* UCSF Chimera--a visualization system for exploratory research and analysis. *J. Comput. Chem.* **25**, 1605-1612, doi:10.1002/jcc.20084 (2004).

12 Emsley, P., Lohkamp, B., Scott, W. G. & Cowtan, K. Features and development of Coot. *Acta Crystallogr. D Biol. Crystallogr.* **66**, 486-501, doi:10.1107/s0907444910007493 (2010).

13 Adams, P. D. *et al.* PHENIX: a comprehensive Python-based system for macromolecular structure solution. *Acta Crystallogr. D Biol. Crystallogr.* **66**, 213-221, doi:10.1107/s0907444909052925 (2010).

14 Goddard, T. D. *et al.* UCSF ChimeraX: Meeting modern challenges in visualization and analysis. *Protein Sci.* **27**, 14-25, doi:10.1002/pro.3235 (2018).

15 DeLano, W. L. *The PyMOL Molecular Graphics System*, <http://www.pymol.org> (2002).

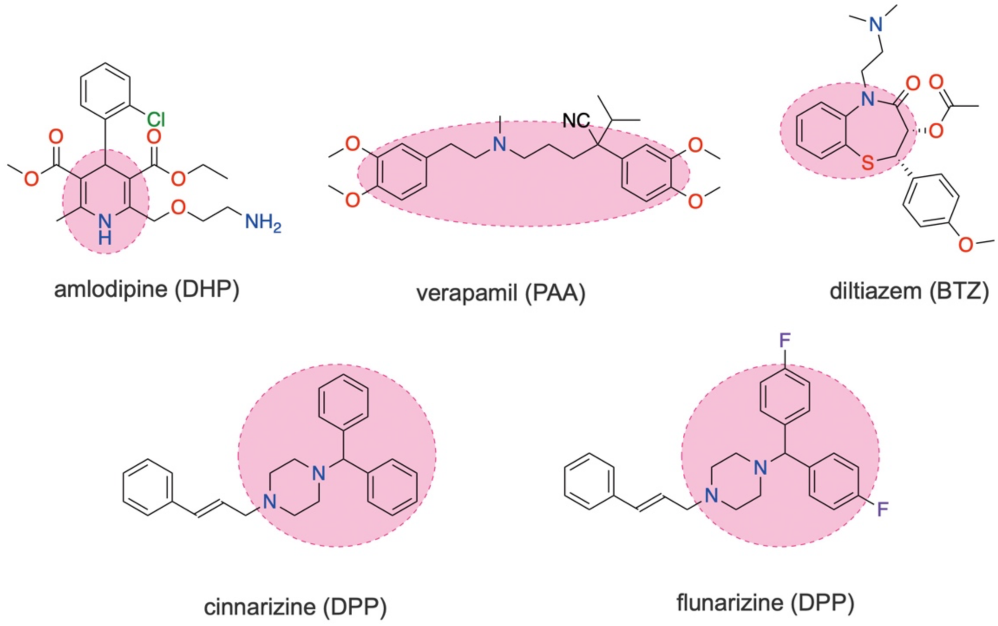

Fig. S1 Chemical structures of the representative drugs targeting L-type Ca_v channels.

Shaded are the core groups. DHP: dihydropyridines; PAA: phenylalkylamines; BTZ: benzothiazepines; DPP: diphenylmethylpiperazines.

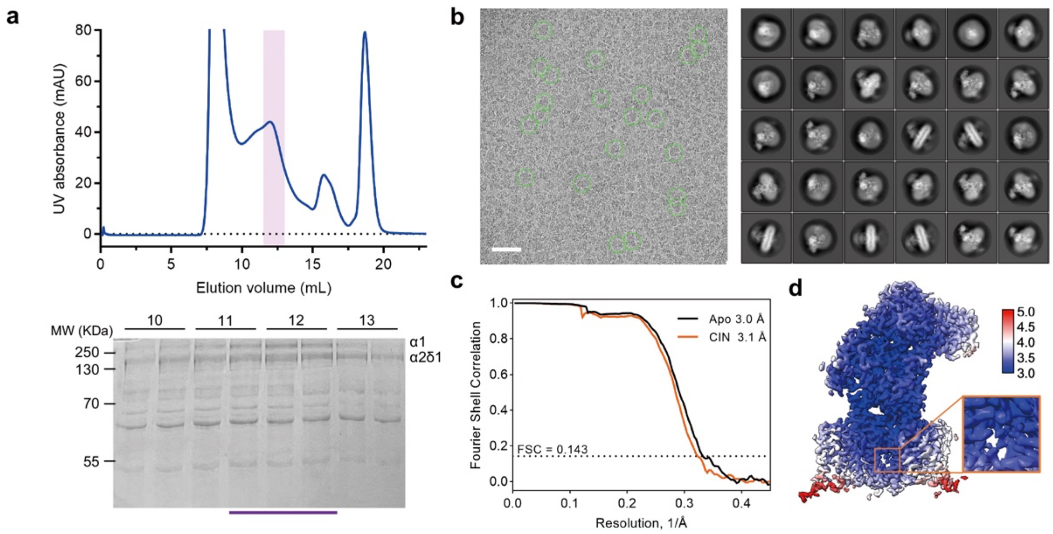

Fig. S2 Structural determination of human Ca$_v$1.3 complex. a Last-step purification of Ca$_v$1.3. *Top*: representative size-exclusion chromatography for proteins obtained from 8 L transfected HEK293F cells. *Bottom*: SDS-PAGE stained by Coomassie-blue. Shaded and underscored peak fractions were used for cryo-sample preparation. **b** Representative cryo-EM micrograph (*left)* and final 2D classification (*right*). The green circles indicate particles in distinct orientations. The box size for the 2D classification is 320 Å. Scale bar, 50 nm. **c** The gold-standard Fourier Shell Correlation (FSC) curves for the overall 3D reconstructions. **d** Heat map for local resolutions of cinnarizine-bound Ca$_v$1.3. Inset shows the densities for cinnarizine and surrounding residues. The map, calculated in Relion, was generated in ChimeraX.

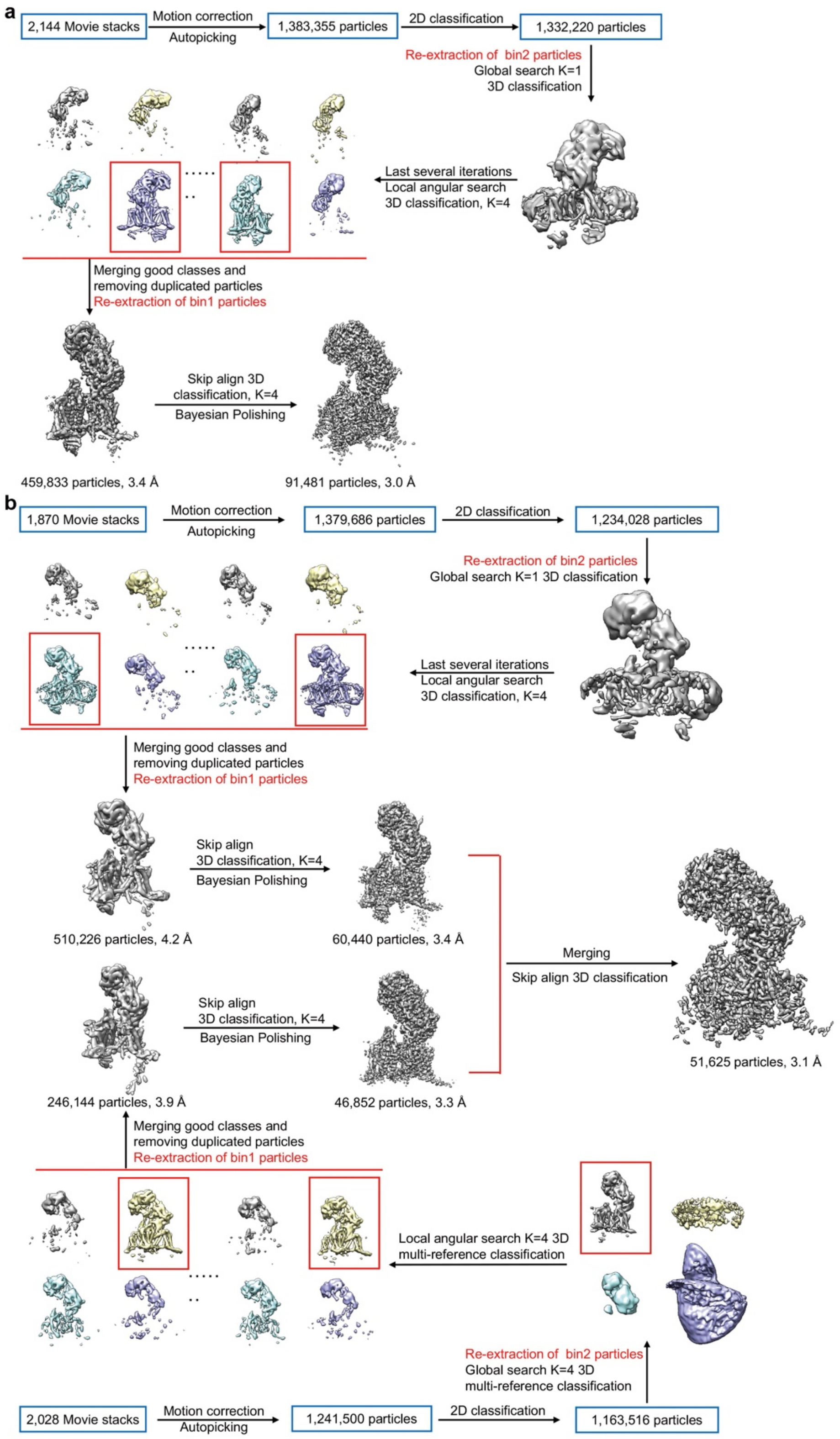

Fig. S3 Cryo-EM data processing for apo (a) and cinnarizine-bound Ca_v1.3 (b). Because no cp-PYT density was observed in final reconstruction, we described this structure as apo state. For Ca_v1.3-cinnarizine structure determination, two batches of datasets were collected.

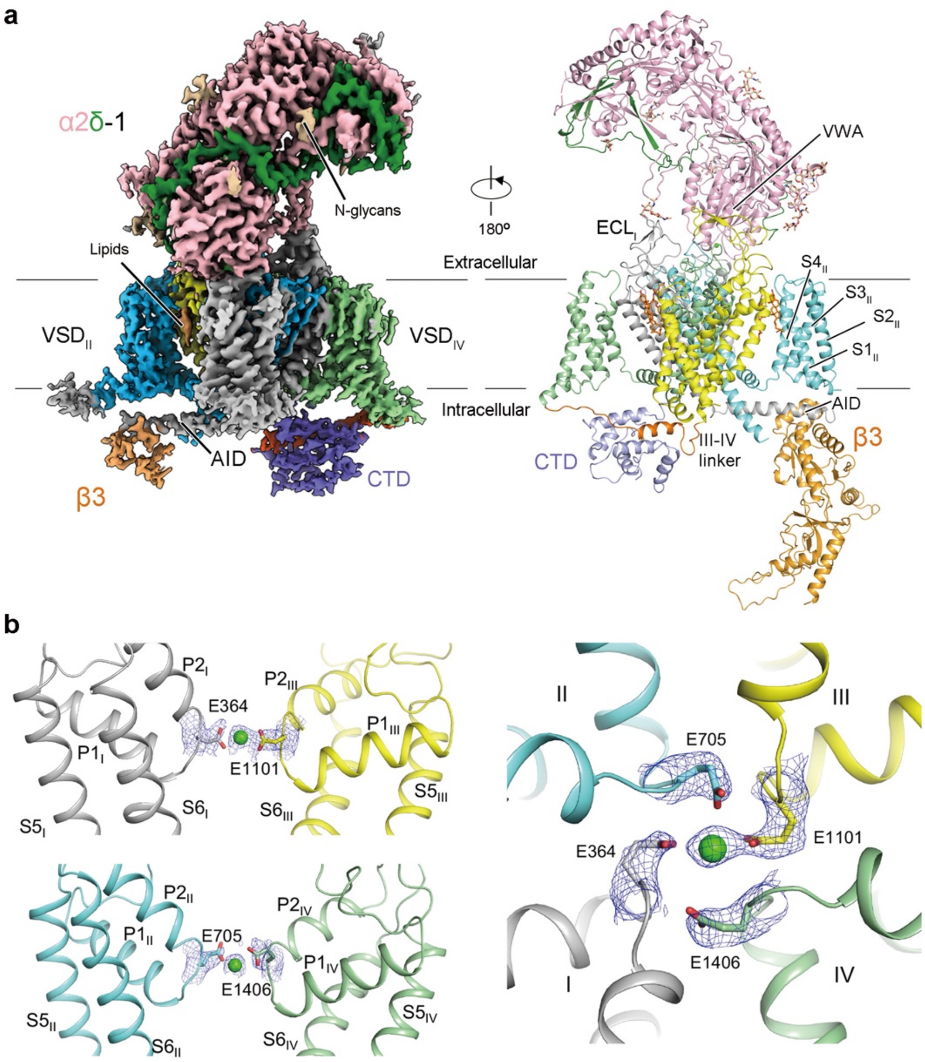

Fig. S4 Cryo-EM structures of human Ca$_v$1.3 complex. a Model building for apo Ca$_v$1.3. The 3D EM map (*left*) and complex structure (*right*) were prepared with the same color scheme. β3 subunit was docked into Ca$_v$1.3 EM map as a rigid body based on the reported Ca$_v$2.2 structure (PDB code 7MIY)[6]. Apart from the polypeptides, seventeen glycans (wheat sticks) and four lipids (orange sticks) were resolved and assigned. VSD, voltage-sensing domain (domain-colored); AID, α1-interacting domain; CTD, C-terminal domain (purple); ECL, extracellular loop. Two Ca^{2+} ions, one in the von Willebrand factor type A (VWA) domain of α2, and the other in the selectivity filter of α1, are shown as green spheres. **b** The selectivity filter vestibule in apo Ca$_v$1.3. Two side views (*left*) and an extracellular view (*right*) are shown. The densities for Ca^{2+} ion and surrounding residues, shown as blue meshes, are contoured at 5 σ in PyMol.

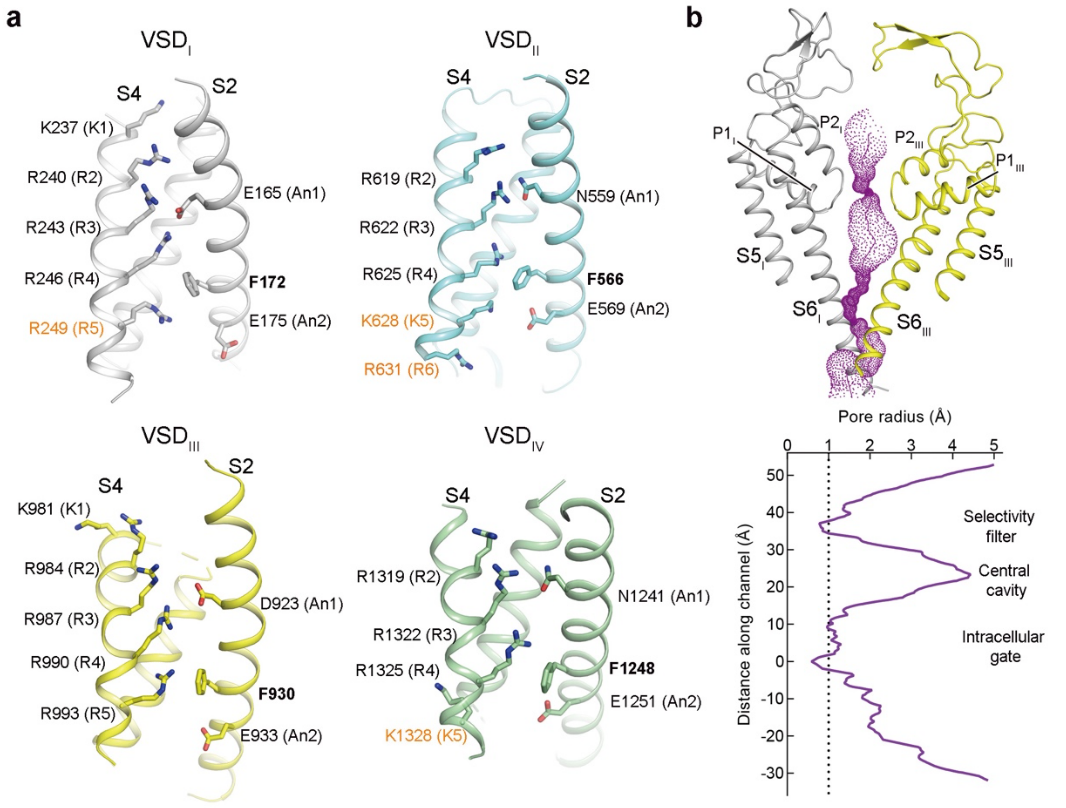

Fig. S5 An inactivated conformation of Ca$_v$1.3. a Four voltage-sensing domains (VSDs) exhibit depolarized ('up') conformations. The gating charge (GC) residues on S4 and their coordinating residues on S2 are shown as sticks. To make the numbering of the GC residues consistent, the GC residue on the first helical turn of S4 is designed as K/R1. The labels for the occluding Phe on S2 are bold. The GCs above and below the occluding Phe are labeled black and orange, respectively. An1 and An2, conserved acidic or polar residues on S2. **b** Closed pore of apo Ca$_v$1.3. The ion permeation path is illustrated by purple dots at the *top*, and the calculated pore radii by HOLE is at the *bottom*. Vertical dashed line marks the radius at 1 Å.

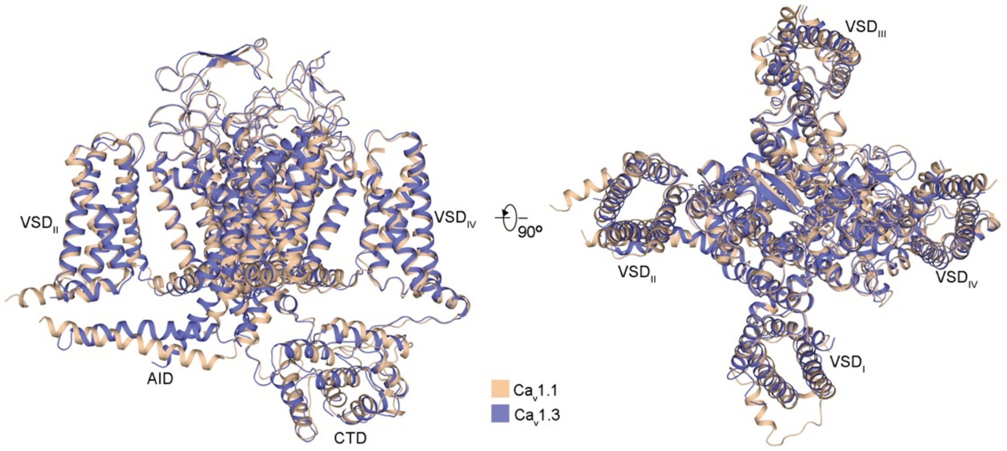

Fig. S6 Structural comparison of the α1 subunits in rabbit $Ca_v1.1$ (PDB code: 5GJV) and human $Ca_v1.3$.

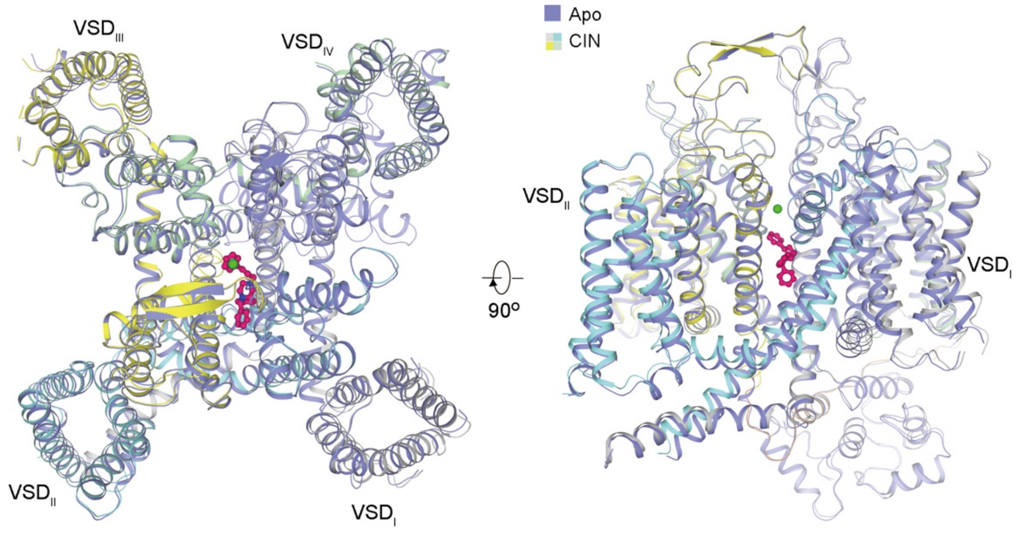

Fig. S7 Nearly identical α1 structures in apo and cinnarizine-bound Ca_v1.3. Two perpendicular views of the superimposed structures are shown. Apo Ca_v1.3 is colored purple, and the drug complex is colored by domain.

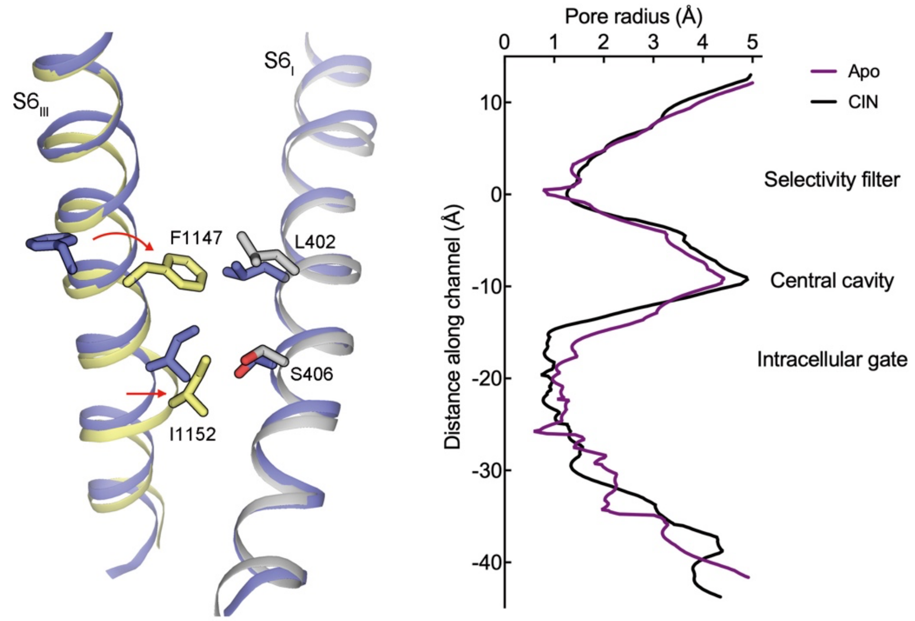

Fig. S8 Structural shift of S6$_{III}$ upon cinnarizine binding tightens the intracellular gate. *Left:* Gating residues on S6$_{I}$ and S6$_{III}$ are shown as sticks. Red arrows indicate the structural shift from apo (colored purple) to cinnarizine-bound Ca$_v$1.3 (grey and yellow). *Right:* Pore radii of apo (purple line) and cinnarizine-bound bound Ca$_v$1.3 (black line) were calculated by HOLE.

Table S1. Statistics for data collection and structural refinement

	Apo-Ca$_v$1.3 (EMD-26514) (PDB 7UHG)	Ca$_v$1.3-CIN (EMD-26513) (PDB 7UHF)
**Data collection and processing**		
Magnification	105,000	105,000
Voltage (kV)	300	300
Electron dose (e-/Å^2)	50	50
Defocus range (μm)	-2.1~-1.9	-2.1~-1.9
Pixel size (Å)	1.114	1.114
Symmetry	C1	C1
Initial particle images (no.)	1,383,355	2,621,186
Final particle images (no.)	91,481	51,625
Map resolution (Å)	3.0	3.1
FSC threshold	0.143	0.143
**Refinement**		
Initial model used (PDB code)	5GJV, 7MIY	Apo Ca$_v$1.3
Model resolution (Å)	3.1	3.3
FSC threshold	0.5	0.5
Map sharpening *B* factor (Å^2)	-64	-65
Model composition		
Non-hydrogen atoms	20416	20381
Protein residues	2502	2502
Ligands	23	21
B factors (Å^2)		
Protein	81.74	54.43
Ligand	67.82	49.34
R.m.s deviations		
Bond lengths (Å)	0.003	0.003
Bond angles (°)	0.553	0.629
Validation		
MolProbity score	1.78	2.18
Clashscore	8.56	9.91
Poor rotamers (%)	0.41	2.31
Ramachandran plot		
Favored (%)	95.43	94.38
Allowed (%)	4.49	5.50
Disallowed (%)	0.08	0.12
